# Limited *Plasmodium* sporozoite gliding motility in the absence of TRAP family adhesins

**DOI:** 10.1186/s12936-021-03960-3

**Published:** 2021-10-30

**Authors:** Konrad Beyer, Simon Kracht, Jessica Kehrer, Mirko Singer, Dennis Klug, Friedrich Frischknecht

**Affiliations:** 1 Integrative Parasitology, Center for Infectious Diseases, Heidelberg University Medical School, Im Neuenheimer Feld 344, 69120 Heidelberg, Germany; 2 German Center for Infection Research, Partner Site Heidelberg, 69120 Heidelberg, Germany; 3 Present Address: Experimental Parasitology, Faculty of Veterinary Medicine, Ludwig-Maximilians-University Munich, Lena-Christ-Straße 48, Planegg, 82152 Munich, Germany; 4 Present Address: Université de Strasbourg, CNRS UPR9022, INSERM U963, Institut de Biologie Moléculaire et Cellulaire, 67000 Strasbourg, France

**Keywords:** *Anopheles*, Transmission, Migration, Malaria, Adhesion

## Abstract

**Background:**

*Plasmodium* sporozoites are the highly motile forms of malaria-causing parasites that are transmitted by the mosquito to the vertebrate host. Sporozoites need to enter and cross several cellular and tissue barriers for which they employ a set of surface proteins. Three of these proteins are members of the thrombospondin related anonymous protein (TRAP) family. Here, potential additive, synergistic or antagonistic roles of these adhesion proteins were investigated.

**Methods:**

Four transgenic *Plasmodium berghei* parasite lines that lacked two or all three of the TRAP family adhesins TRAP, TLP and TREP were generated using positive–negative selection. The parasite lines were investigated for their capacity to attach to and move on glass, their ability to egress from oocysts and their capacity to enter mosquito salivary glands. One strain was in addition interrogated for its capacity to infect mice.

**Results:**

The major phenotype of the TRAP single gene deletion dominates additional gene deletion phenotypes. All parasite lines including the one lacking all three proteins were able to conduct some form of active, if unproductive movement.

**Conclusions:**

The individual TRAP-family adhesins appear to play functionally distinct roles during motility and infection. Other proteins must contribute to substrate adhesion and gliding motility.

**Graphical Abstract:**

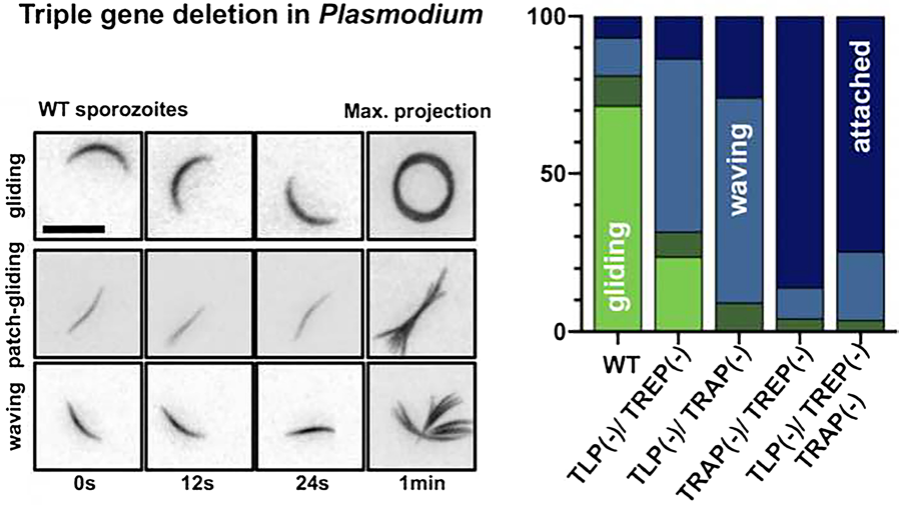

**Supplementary Information:**

The online version contains supplementary material available at 10.1186/s12936-021-03960-3.

## Background

*Plasmodium* sporozoites are the most versatile forms of the malaria parasite [1]. They develop within oocysts at the mosquito midgut and escape from these into the haemolymph of the mosquito [2, 3]. Within the haemolymph, sporozoites are transported passively throughout the body cavity of the mosquito [4–6] and enter into the mosquito salivary glands. There sporozoites accumulate in large non-motile clusters while individual sporozoites progress slowly into the salivary duct [7–9]. During the bite of the mosquito 10–100 sporozoites are transmitted to the host [9–13]. Sporozoites, similar to most arthropod transmitted pathogens [14], are deposited in the dermis of the skin [6, 12, 15–21]. Within the dermis, sporozoites of avian parasites can already enter host cells and differentiate [22]. Sporozoites of mammalian infecting parasites have been shown to migrate at high speed exceeding 1 µm per second, which strikingly is an order of magnitude faster than neutrophils [18, 23]. Sporozoites migrate through cells in the dermis, a capacity that is essential for entering the circulatory system [24, 25]. Sporozoites can enter both blood or lymph vessels [18] and those entering the blood are transported throughout the body to reach the liver [26, 27]. In the liver sporozoites enter and differentiate within hepatocytes into red cell infecting merozoites [25, 28, 29].

**Fig. 1:**
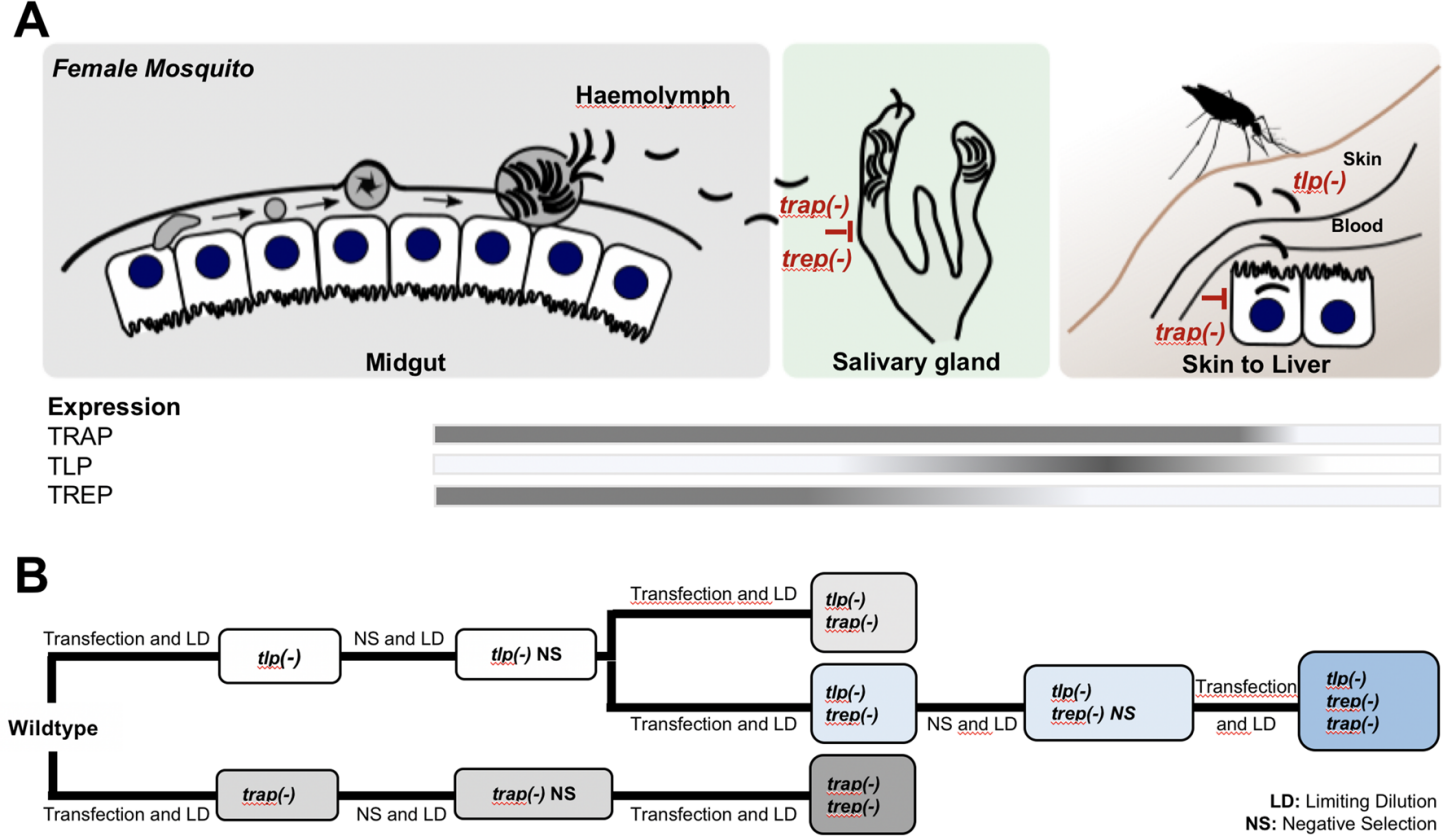
Generation of parasite lines lacking multiple TRAP family adhesins. **A** Overview cartoon showing the effect of single knockouts of TRAP, TLP or TREP on *Plasmodium* sporozoite live cycle progression. *trap(-)* parasites are nearly completely blocked in salivary gland colonization. *trep(-)* parasites show a severe reduction in salivary gland colonization, but those that get in can progress normally. *tlp(-)* show no defect in salivary gland colonization but a small defect in migration in the skin. *trap(-)* parasites isolated from oocysts also cannot enter into the liver. Bars show expression of the different genes. **B** Flow chart illustrating the transgenesis strategy used to generate double and triple knockout parasites

To achieve this remarkable journey, sporozoites employ a set of proteins that are specifically expressed only in sporozoites. The first protein that was shown to play a role in salivary gland invasion was the thrombospondin-related anonymous protein (TRAP). A parasite line lacking the *trap* gene still formed sporozoites within oocysts but these were largely incapable of entering salivary glands [30, 31]. Haemolymph-derived sporozoites lacking *trap* were further incapable of performing productive motility [30] and showed a partial defect in substrate adhesion [32]. However, adhesive *trap(-)* sporozoites were still capable of performing a back-and-forth type of motility termed patch-gliding, which is typically found only in sporozoites isolated from the haemolymph [32]. Normal gliding motility of sporozoites, of other motile *Plasmodium* stages, and of related apicomplexan parasites is driven by an actin-myosin based motor located underneath the plasma membrane of the parasite and TRAP or TRAP-related proteins couple this motor to the extra-cellular substrate [33–36]. TRAP-related and TRAP family proteins share a similar domain structure spanning the plasma membrane [3]. They contain extracellular adhesive domains and they share a short cytoplasmic tail that is thought to couple the proteins to actin filaments [37]. Apart from TRAP, two more TRAP family members are expressed by sporozoites: the TRAP-like protein TLP and the TRAP-related extraordinary protein TREP, also named S6 or UOS3 [38–41] (Fig. 1A). Sporozoites lacking *tlp* show only a slight phenotype in migration within the skin and hence some delayed infectivity in mice [38, 39, 42]. In contrast, sporozoites lacking *trep* have a strongly reduced capacity to enter salivary glands, but those that do enter the salivary gland subsequently infect mice normally [41]. Experiments employing optical traps to measure the forces that sporozoites can exert suggest that *trap(-)*, *trep(-)* and *tlp(-)* sporozoites are all partially deficient in force generation [43, 44]. Intriguingly, the cohesive strengths of substrate adhesion sites are distinct in parasites lacking *trap* and *trep* [43].

Investigation of salivary gland sporozoites lacking *tlp* indicates that TLP organizes macromolecular assemblies in order to couple the retrograde flow of actin filaments to force production [44, 45]. TRAP is expressed early on in midgut derived sporozoites and still present and important for salivary gland sporozoites, while TREP expression wanes in the salivary glands although the protein is still present at this stage [40]. In contrast, TLP expression only starts after salivary gland entry [39] (Fig. 1A). Currently, it is not clear whether the individual TRAP family members exert their effects individually or as an ensemble. Here, to address possible additive, synergistic, or antagonistic effects of the three proteins, and to investigate if sporozoite motility only depends on TRAP family adhesins, three parasite lines were generated and investigated that lacked two of the TRAP family members and one parasite line lacking all three.

## Methods

### Handling of parasite lines

Parasite lines were generated in blood stages and maintained in Swiss or NMRI mice through transfer of 10,000 blood stage parasites into the tail vein of naïve mice. Where possible (wild type and some mutants) parasites were cycled through mosquitoes prior to generating frozen aliquots. To this end, *Anopheles stephensi* mosquitoes were allowed to bite anesthetised mice, which contained gametocytes in their blood. Three weeks after infection two naïve mice were bitten by the infected mosquitoes and blood stage parasitaemia was followed by daily blood smears. Whole blood was harvested by cardiac puncture. New infections were started by intraperitoneal injection of thawed aliquots.

### Generation of plasmids and parasite lines

All generated plasmids were based on either the Pb238 or the Pb301 transfection vector [46, 47] containing the 5′ and 3′ UTRs of *tlp* or *trep* as well as the gene encoding the green fluorescent protein (GFP) [48]. All primer sequences are listed in Additional file 5: Table S1. Prior to transfection, all vectors were linearized with *SacII* and *PmeI.* Transfections were done as described in Janse et al. [49]. Schizonts were purified using a 55% nycodenz cushion followed by electroporation using the Amaxa T-cell Nucleofector kit. Parasites were maintained in 6–8 week old NMRI or CD1 mice provided with drinking water supplemented with pyrimethamine until a parasitaemia of more than 2% was reached.

*Pb262* transfection vector [3, 50]: This vector uses the hDHFR-yFCU selection marker cassette [51] and the reporter gene mCherry under the control of the *csp* promoter. Using the Pb238 as parental vector, the selection marker cassette was replaced with the *dhfr *3′UTR–*ef1α* 5′UTR–*hDHFR*–*yFCU*–*dhfr* 3′UTR from PlasmoGEM transfection vector [52], which was amplified with P600 and P601 and cloned with *EcoRV HindIII* into Pb238. Next, the 5′UTR of *csp* was amplified from *P. berghei* gDNA with primers P207 and P208 and cloned into the vector with *EcoRI* and *NdeI*. The open reading frame of the red fluorescent protein encoding gene *mCherry* was amplified with primers P238 and P232 and inserted into the vector with *NdeI*/*BamHI*. The resulting vector is termed Pb262.

*trap(-)* and *trap(-)NS*: For the generation of *trap(-)* parasites the plasmid PbGEM-107890 was requested from PlasmoGEM [53, 54]. It replaces most of the TRAP coding sequence with the positive–negative selection marker *hDHFR-yFCU*. For negative selection [55] the drinking water of mice was supplemented with 1 mg/ml 5-FC. The parasite line was already generated and characterized in a previous study [31] and used here as basis to generate the double mutant.

*trap*(-)/*trep*(-)/*tlp(-)mCherry*: The final TLP-KO transfection vector (PbCSmCherryYFCU_TLP-KO) was generated by amplifying the 5′ UTR of *tlp* from *Plasmodium berghei* gDNA with primers P159 and P160 and cloning into Pb238 via SacII/ NotI. The *tlp* 3′ UTR was amplified with primers P161 and P162 and then cloned via *HindIII*/*KpnI*. Next, using *HindIII* and *NotI* restriction sites of the ‘TLP plasmid’ and Pb262, the selection cassette and the *mCherry* reporter gene of the Pb262 plasmid was inserted to create the final TLP-KO transfection plasmid as illustrated in Additional file 1: Fig. S1.

tlp(-)mCherryNS: For negative selection [55] the drinking water of mice infected with *tlp(-)mCherry* parasites was supplemented with 1 mg/ml 5-FC. Subsequently clonal parasites which lost the resistance cassette by homologous recombination were obtained by limiting dilution.

*tlp(-)mCherry|trap(-)*: The PlasmoGem vector PbGEM-107890 [53, 54] was transfected into selection marker free *tlp(-)mCherry* parasites. Clonal parasites were obtained by limiting dilution. Genotyping was performed as described in Additional file 1: Fig. S1.

*tlp(-)mCherry|trep(-)*: In order to remove *trep* in the *tlp(-)mCherry*NS parasites, the 5′ UTR of *trep* was amplified with primers P100 and P101, and cloned into Pb238 via *SacII* / *NotI*. The 3′ UTR was amplified with primers P102 and P103 and then cloned via *HindIII*/*KpnI*. The resulting vector and Pb262 were digested with *HindIII* and *NotI* to insert the CSPmCHerryYFCU cassette, yielding PbCSmCherryYFCU_TREP-KO. This vector was used to create *trap(-)|trep(-)mCherry* (see below). For two of the planned knockout lines that already expressed mCherry in the *tlp* locus [TLP(-)/TREP(-) and TLP(-)/TRAP(-)/TREP(-)], a TREP(-) transfection vector [PbYFCU_TREP(-)] was created that lacked the *mCherry* reporter gene. In order to remove the mCherry reporter gene and the *CSP* promoter, PbCSmChYFCU_TREP-KO was digested with *NotI* and *EcoRV*, blunted to get rid of any overhangs and finally re-ligated.

*tlp(-)mCherry|trep(-)NS*: The vector PbYFCU_TREP-KO was transfected into selection marker free *tlp(-)mCherry* parasites. Clonal parasites were obtained by limiting dilution. For negative selection [55] the drinking water of mice infected with *tlp(-)mCherry|trep(-)* parasites was supplemented with 1 mg/ml 5-FC. Subsequently, clonal parasites which lost the resistance cassette by homologous recombination were obtained by limiting dilution. Genotyping was performed as described in Additional file 2: Fig. S2.

*trap(-)|trep(-)mCherry*: The PbCSmChYFCU_TREP-KO transfection vector was transfected into *trap(-)NS* parasites. Clonal parasites were obtained by limiting dilution. Genotyping was performed as described in Additional file 3: Fig. S3.

*tlp(-)mCherry|trep(-)|trap(-)*: The PlasmoGem vector PbGEM-107890 [53, 54] was transfected into selection marker free *tlp(-)mCherry|trep(-)* parasites. Clonal parasites were obtained by limiting dilution. Genotyping was performed as described in Additional file 4: Fig. S4.

### Cryopreservation of parasites

For longterm storage 100 µl of blood containing parasites together with 200 µl of freezing solution (10% glycerol in Alsvers solution) was kept in liquid nitrogen.

### Isolation of parasites and gDNA preparation

To isolate parasites for genotyping, at least 500 µl of blood from an infected mouse was harvested via cardiac puncture. After the addition of 1 ml PBS, red blood cells were lysed using 150 µl of 1% saponin/ PBS. After centrifugation, the resulting parasite pellet was washed once with 1 ml PBS and resuspended with 200 µl PBS. Genomic DNA was isolated using a qiagen blood and tissue kit according to the manufacturers protocol.

### Mosquito infection

Per infected mosquito cage, 20 million bloodstage parasites were transferred by intraperitoneal injection into 2 naïve NMRI or CD1 Swiss mice. After continued parasite growth for 3–5 days, the blood of the mice was monitored for the presence of gametocytes as estimated from observed exflagellation events. To do so, a drop of blood from the tail vein was placed on a glass slide and covered with a cover slip. After an incubation period of 10 min at room temperature, the slide was examined under a light microscope (Zeiss Axiostar, 100 × objective; phase contrast). Three or more exflagellation events per field of view were deemed sufficient for subsequent mosquito infection.

To infect mosquitoes, mice were anesthetized with 100 µl ketamine/xylazine (100 mg/ml ketamin + 20 mg/ml xylazine) and placed on top of a cage containing about 300 *An. stephensi* mosquitoes that were starved for at least 4 h. Mosquitoes were allowed to bite infected mice for about 20 min and were then transferred into an incubator (T: 21 °C; 80% humidity) for parasite development.

### Mosquito dissection

To obtain midguts, haemolymph, and salivary glands, infected mosquitoes were dissected under an SMZ 1500 binocular microscope (Nikon) using two syringes with needles. For the dissection of midguts, the last two abdominal segments were cut off and the remaining abdomen was detached from the thorax to expose the midgut, which was subsequently transferred into a reaction tube containing/filled with 200 µl phosphate buffered saline (PBS). Salivary glands were obtained by carefully decapitating the mosquito with a pulling movement in a way that left the salivary glands attached to the head. Subsequently the salivary glands were separated from the head and transferred to a reaction tube supplied with 200 µl PBS. To collect haemolymph the last two abdominal segments of the mosquito were cut off and the thorax pierced with a finely drawn pasteur pipette filled with PBS. The mosquito was gently flushed with PBS and the drops forming at the abdominal opening were collected on a piece of Parafilm. Drops were then transferred into an empty reaction tube using a pipette.

### Staining and counting of oocysts

Oocyst numbers were determined between day 12 and 17 post infection. Midguts were isolated and incubated in 100 µl PBS supplemented with 1% NP40 for 20 min at room temperature. Staining of oocysts was performed using 100 µl PBS supplemented with 0.1% Mercurochrome. Midguts were incubated for up to 2 h. After staining, the midguts were washed three times with PBS, transferred to a glass slide with a pasteur pipette and covered with a cover glass. Oocysts were counted manually with an Axiovert 200 M epifluorescence microscope (Carl Zeiss) using transmission light, a green filter and an 10 × air objective.

### Counting of sporozoites

Midgut sporozoites were counted on days 14 and 16 after infection, haemolymph sporozoites on day 14–16 and salivary gland sporozoites on day 17. To determine sporozoite numbers, salivary glands and midguts were collected from at least 10 mosquitos and smashed with a plastic pestle. All samples were counted under a light microscope with a 40 × air objective using a haemocytometer (Neubauer improved) and phase contrast.

### Sporozoite gliding assays

Haemolymph sporozoite gliding assays were performed on day 14 post infection and salivary gland sporozoite gliding assays on day 17 post infection. Haemolymph and salivary glands were collected as described above. To free sporozoites from salivary glands, glands were smashed using a plastic pestle before gliding assays were performed. Both haemolymph and salivary gland samples were centrifuged, the supernatant was discarded and sporozoite pellets were resuspended in 100 µl 3% BSA/RPMI solution to activate gliding motility. Resuspended sporozoites were transfered to a glass bottom 96-well plate and centrifuged at 1000 rpm for 3 min at room temperature. Imaging was done with an Axiovert 200 M epifluorescence microscope using a 25 × glycerin objective. A series of timelapse videos with one frame per 3 s was obtained using a Prime BSI camera (Photometrics) mounted on the sideport of the microscope. Videos were analysed with Fiji (ImageJ). Sporozoites were classified in one of five gliding patterns (gliding, patch gliding, waving, attached, and floating) and sporozoite speed was determined using the ImageJ plugin ‘Manual Tracking’ [56].

### Transmission experiments

To check the infectivity of the generated KO parasites, naive C57BL/6 mice were either exposed to infected mosquitoes or injected intravenously (i.v.) with salivary gland or haemolymph sporozoites. For infection of mice by bite, naive C57BL/6 mice were anesthetized with 100 µl ketamin/xylazin and then placed on top of net-covered cups containing 10 mosquitoes each. Mice were left on cups for around 20 min. Blood-filled mosquitoes were subsequently dissected and salivary glands were collected to determine sporozoite numbers per mosquito. For i.v. injection, haemolymph and salivary gland sporozoites were collected and counted as described above. 10,000 haemolymph or 100 salivary gland sporozoites were injected into the tail vein of naive C57BL/6 mice. For both bite back and *i.v.* transmission experiments, parasitaemia was monitored through daily blood smears stained with Giemsa solution, starting on day 3 post infection. Mice were sacrificed on day 10 after infection at the latest to minimize suffering due to cerebral malaria and anaemia.

### Ethics statement

All animal experiments were performed according to FELASA guidelines and were officially sanctioned by the responsible German authorities (Regierungspraesidium Karlsruhe) in accordance with the German Animal Welfare Act (Tierschutzgesetz). Female NMRI or CD1 swiss mice, ordered from JANVIER or Charles River Laboratories, were used for rearing of the parasites and infection of mosquitoes. Transmission experiments were performed using female C57BL/6 mice, ordered from Charles River Laboratories.

### Statistical analysis

Statistical analyses were performed using GraphPad Prism 6.0 (GraphPad, San Diego, CA, USA). A value of p < 0.05 was considered significant. Specifically used tests are indicated in the figure legends.

## Results

### Generation of *Plasmodium berghei* parasite lines lacking two and three TRAP family adhesins

In order to generate parasite lines lacking more than one TRAP family gene, a strategy based on sequential positive and negative selection [51] was employed (Fig. 1B). Vectors for deletion of *trep* and *tlp* were engineered to replace the endogenous gene with the gene encoding for the red fluorescent protein mCherry controlled by the circumsporozoite protein (*csp*) promoter, which is strongly expressed throughout the life of sporozoites (Additional file 1: Fig. S1). The vector for the deletion of *trap* was obtained from PlasmoGEM [53, 54, 57], which also contained a cassette enabling positive–negative selection [31]. A clonal *tlp* knockout line was generated and the drug resistance cassette was recycled by negative selection [31]. Subsequently, vectors targeting the *trap* and *trep* genes were transfected into the *tlp(-)* line (Additional file 1: Fig. S1, Additional file 2: Fig. S2). The *trep* KO vector was additionally transfected into *trap(-)* parasites that were generated previously yielding the line *trep*(-)/*trap*(-) [31] (Additional file 3: Fig. S3). A triple mutant line lacking all three TRAP family adhesins was obtained by negative selection of the *trap(-)/tlp(-)* line followed by deletion of *trep* (Additional file 4: Fig. S4).

### Different developmental arrest of the parasite lines in mosquito midguts

**Fig. 2:**
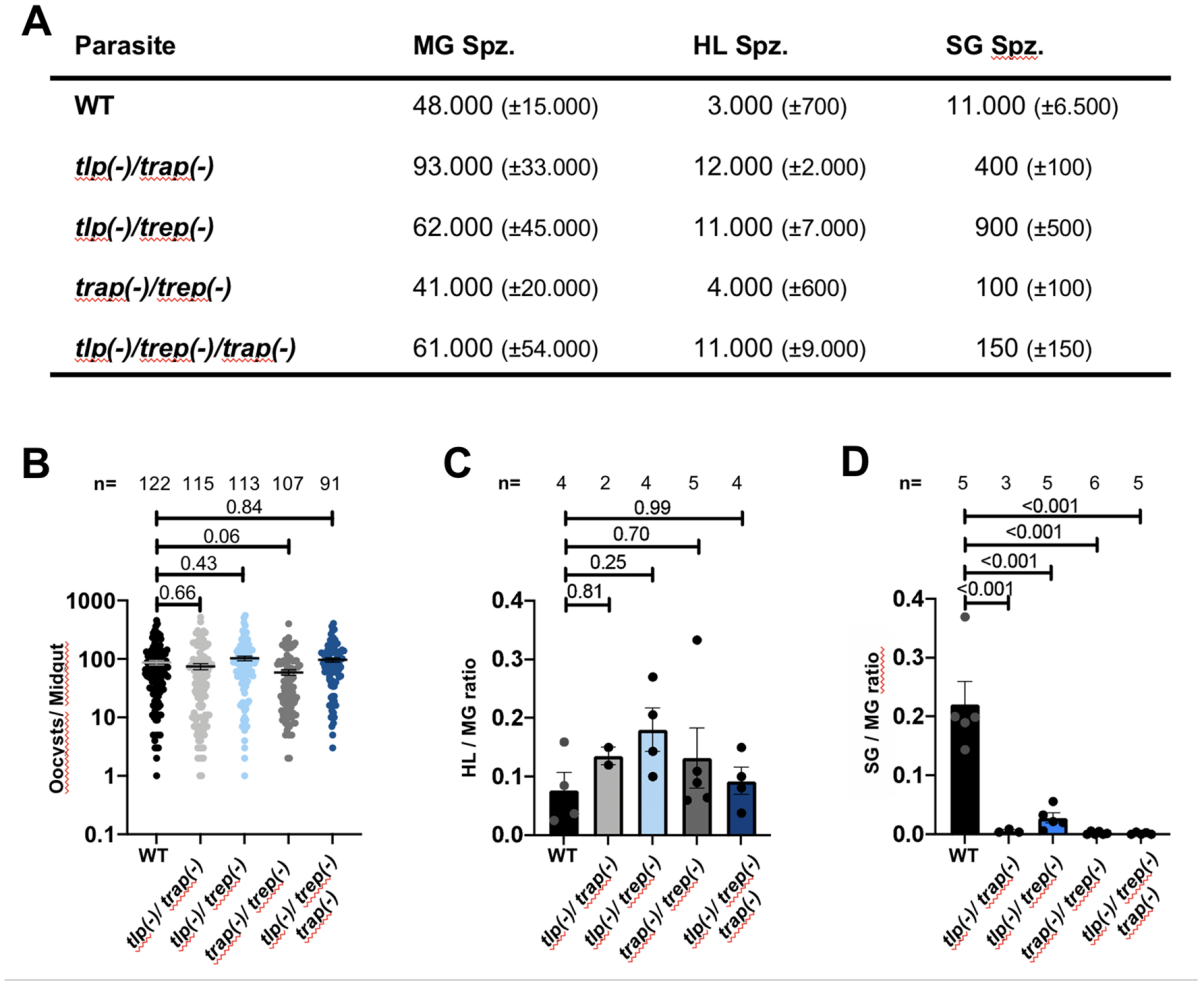
Double and triple deletions can egress from oocysts but have defects in salivary gland entry. **A** Average numbers of midgut (MG), haemolymph (HL) and salivary gland (SG) derived sporozoites per mosquito ± SD. Data from three independent feeding experiments. **B** Oocyst numbers of the different parasite lines. Black lines show mean ± SEM. One-way Anova test with subsequent Dunnetts multiple comparison indicates significance (p-value indicated above bars). Data from 2–4 independent experiments as follows: 2 for *tlp(-)/trap(-)*, 3 for *tlp(-)/trep(-)/trap(-)* and 4 for WT, *tlp(-)/trep(-)* and *trap(-)/ trep(-)*.** C** Ratio of haemolymph to midgut sporozoite numbers for the different parasite lines. Bars show mean ± SEM. One-way Anova test with subsequent Dunnetts multiple comparison indicates significance (p-value indicated above bars). Data from 2 to 5 independent experiments as indicated above the bars.** D** Ratio of salivary gland to midgut sporozoite numbers for the different parasite lines. Bars show mean ± SEM. One-way Anova test with subsequent Dunnetts multiple comparison indicates significance (p-value indicated above bars)

All parasite lines displayed normal growth of blood stages and infected mosquitoes at similar rates leading to comparable numbers of oocysts in mosquitoes (Fig. 2A, B). Accordingly, the numbers of midgut sporozoites varied between cages infected with different mice but were all in the range of what were previously observed in our laboratory (compare range with e.g., [31]) (Fig. 2A). All parasite lines also showed normal to elevated numbers of sporozoites in the haemolymph (Fig. 2A) leading to a midgut-to-haemolymph sporozoite ratio that is comparable to wild type sporozoites (Fig. 2C). However, the numbers of sporozoites in the salivary glands as well as the midgut-to-salivary gland sporozoite ratio varied greatly (Fig. 2A, D). Sporozoite numbers of the *trep(-)/tlp(-)* line were in the range expected for *trep(-)* parasites [40, 41], and all lines lacking *trap* showed very low numbers of salivary gland-resident sporozoites comparable to *trap(-)* sporozoites [30, 31].

### Sporozoites from all parasite lines could perform unproductive gliding

**Fig. 3:**
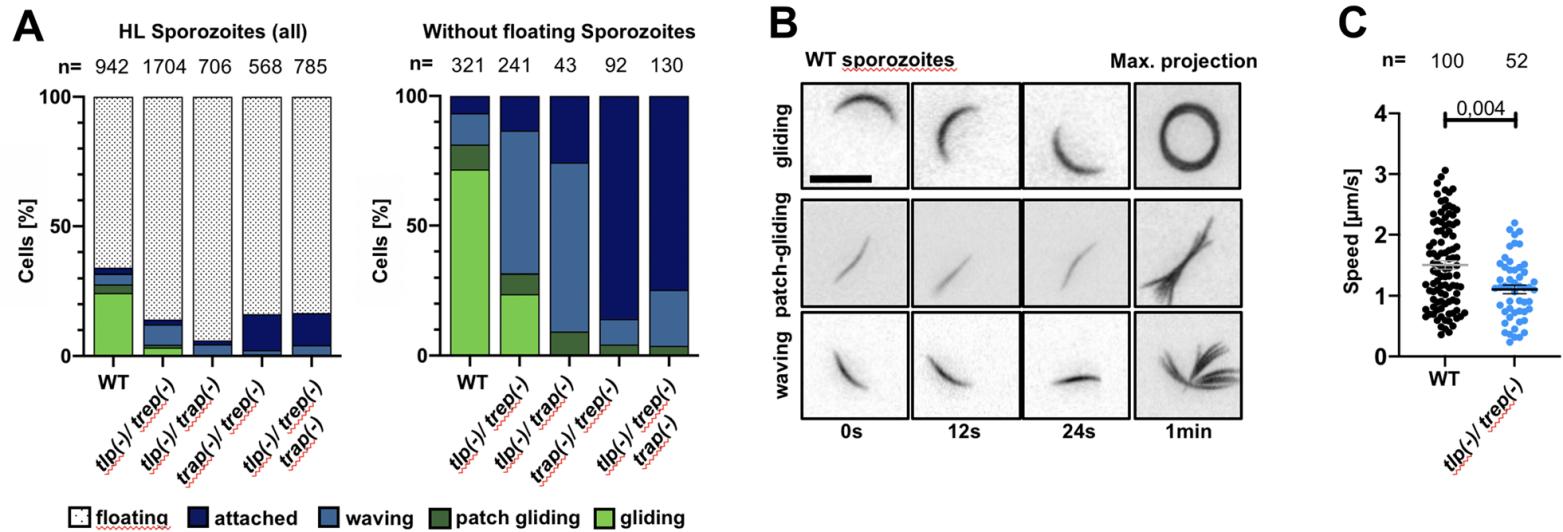
Transgenic lines display weak adhesion and low rates of gliding motility. **A** Distribution of different movement patterns observed in haemolymph sporozoites. Data from three independent infections per line.** B** Example time lapse images of wild type sporozoites undergoing persistent gliding, patch-gliding and waving. Time is indicated below the images. Scale bar: 10 µm. **C** Speed of haemolymph derived sporozoites undergoing persistent gliding motility. Black lines show mean ± SEM. Students t-test indicates significant differences. Data from two different infections per line

Next sporozoites isolated from the mosquito haemolymph were analysed if they could attach to substrates, a prerequisite of gliding motility. This analysis revealed an attachment defect for all knockout lines in comparison to wild type controls (Fig. 3A). While around 35% of control sporozoites attached to glass, only between 10 and 20% of the lines lacking two or three adhesins did so, suggesting that TRAP and TREP, the two TRAP-family adhesins expressed at this stage, contribute to the capacity of the sporozoite to adhere. Subsequently, motility and movement patterns of attached sporozoites were investigated. Three different types of sporozoite movement have been described [32, 58]: (i) productive gliding motility, during which the parasite progresses at high speed on a circular path that is dictated by the curvature of the sporozoite, (ii) patch gliding, during which the parasite moves over a single substrate adhesion point in a back-and-forth manner at peak speeds higher than those of productively gliding sporozoites and (iii) waving, during which sporozoites attach at one end to the substrate and move the parasite body around (Fig. 3A, B).

Quantification of the different movement patterns showed that more *trep(-)/tlp(-)* sporozoites were actively moving compared to the other double knockouts as well as the triple knockout (Fig. 3A). Also *trep(-)/tlp(-)* sporozoites were the only ones undergoing productive gliding motility, albeit at lower frequency and speed than wildtype parasites (Fig. 3A, C). In contrast, *trap(-)/tlp(-)* and *trap(-)/trep(-)* sporozoites failed to undergo productive gliding. All parasite lines exhibited patch-gliding behaviour at slightly different but consistently very low levels (Fig. 3A). Notably the two parasite lines lacking both TRAP and TREP showed much less waving compared to the lines where only one of these adhesion proteins is deleted. The fact that even the triple knockout shows some active motion suggests that other non-TRAP-family proteins are contributing to waving and patch-gliding motion, while TRAP is essential for sporozoites to perform active and directed motility.

**Fig. 4:**
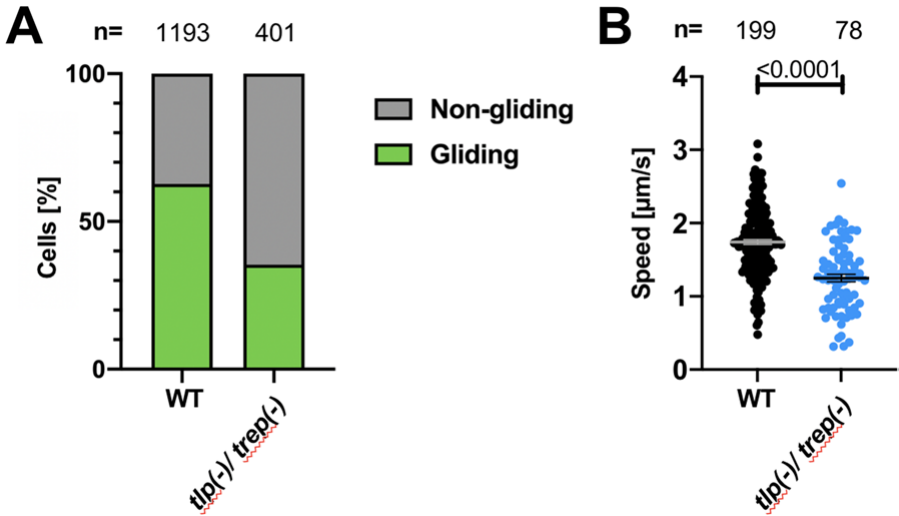
Subtle gliding defect of salivary gland derived *trep(-)/tlp(-)* sporozoites. **A** Percentage of non-gliding and gliding salivary gland derived *trep(-)/tlp(-)* and wild type (WT) sporozoites. **B** Speed of salivary gland (SG) derived sporozoites undergoing persistent gliding motility. Black lines show mean ± SEM. Students *t*-test indicates significant differences. Data from four independent infections per line for **A** and **B**

Next, salivary-gland derived sporozoites were examined from the *trep*(-)/*tlp*(-) line, the only line that demonstrated some productive movement in haemolymph sporozoites. Surprisingly, the rate of persistently gliding *trep(-)/tlp(-)* salivary gland sporozoites was only slightly reduced compared to wildtype controls (Fig. 4A). However, similar to haemolymph *trep*(-)/*tlp*(-) sporozoites, salivary gland *trep*(-)/*tlp*(-) sporozoites moved significantly slower compared to wildtype (Fig. 4B). The observation that *trep*(-)/*tlp*(-) salivary gland sporozoites were capable to adhere suggests that TREP plays a role in adhesion of haemolymph sporozoites but possibly less so in salivary gland sporozoites.

### Transmission to mice is diminished in *trep(-)/tlp(-)* sporozoites

**Fig. 5:**
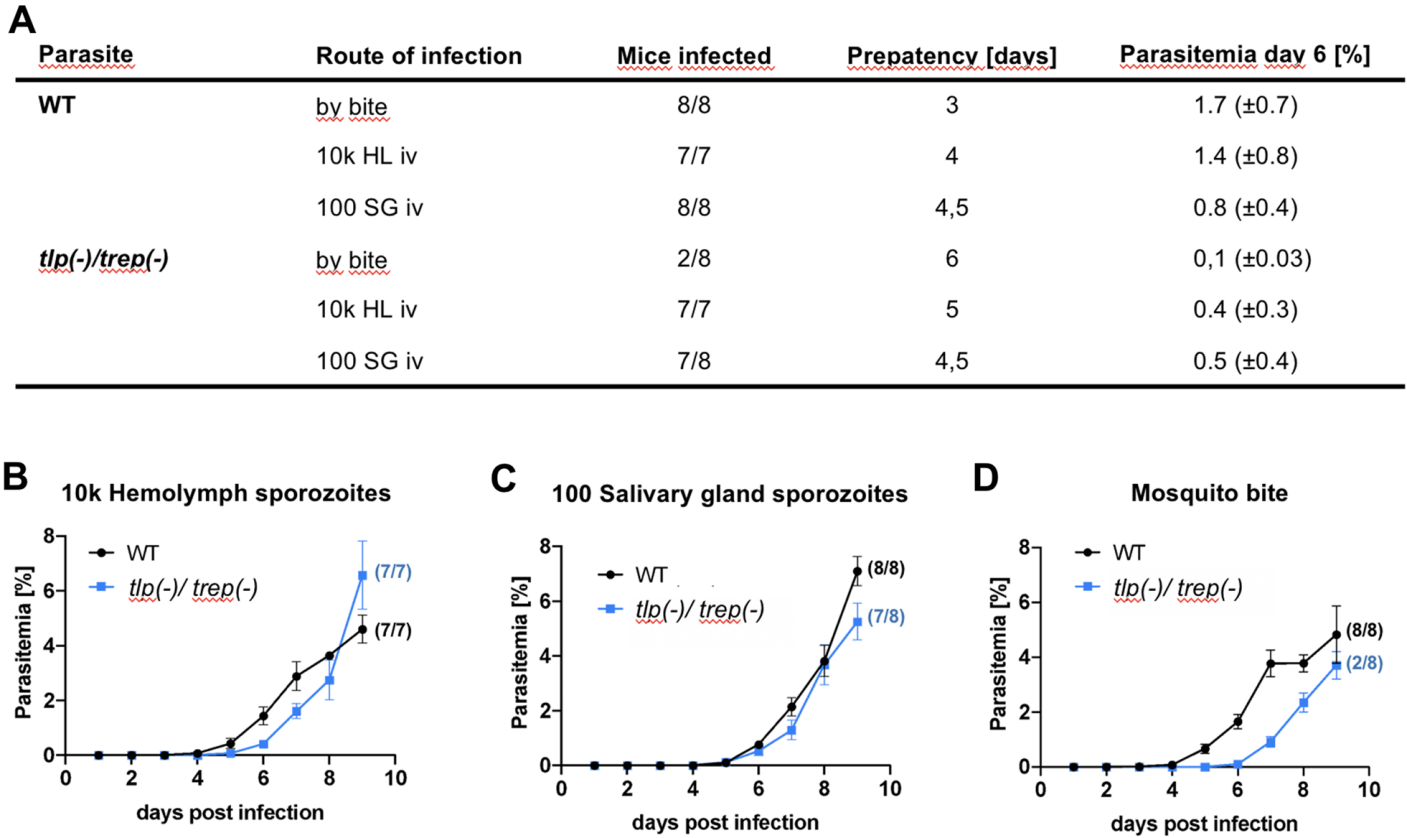
*trep(-)/tlp(-)* sporozoites transmit less efficiently to mice. **A** Summary of the transmission experiments with numbers of infected/total parasite injected (mosquito exposed) mice, prepatency and parasitaemia on day 6 given for the different parasite lines and the respective modes of transmission. Graphs display the development of blood stage parasitaemia in mice infected by intravenous injection of 10.000 haemolymph sporozoites. **B** or 100 salivary gland derived sporozoites. **C** and by the bites of 10 mosquitoes. **D** Wildtype control: black line, *trep(-)/tlp(-)*: blue line; parasitaemia shows average for the two mice that became patent). Error bars indicate SEM

The observation of productive movement in haemolymph and salivary gland sporozoites of the *trep*(-)/*tlp*(-) line suggested that these sporozoites might be able to infect mice. This possibility was tested in two ways: Either equal numbers of haemolymph or salivary gland derived sporozoites from the *trep(-)/tlp(-)* line or the wildtype control were injected intravenously into C57BL/6, or mice were infected by allowing infected mosquitoes to feed (bite-back infection) (Fig. 5). Upon infection with haemolymph-derived *trep*(-)/*tlp*(-) sporozoites all seven infected mice developed blood-stage parasitaemia, yet with a delay of 1 day compared to wild type controls (Fig. 5A, B). This largely mimics the phenotype of the *trep(-)* parasite line [40]. In contrast, infection with just 100 salivary gland derived *trep(-)/tlp(-)* sporozoites showed no delay in blood stage onset or development compared to wildtype controls, although only 7/8 mice injected with *trep(-)/tlp(-)* sporozoites became positive (Fig. 5A, C). Transmission by bite, however, showed a large decrease in transmission efficiency of *trep(-)/tlp(-)* sporozoites infected mosquitoes (Fig. 5A, D). Only 25% (2/8) of mice became infected with *trep*(-)/*tlp*(-) and blood stage parasitaemia was delayed by 3 days compared to wildtype controls (Fig. 5A, D). This is likely a combined effect of the decreased number of salivary gland-resident sporozoites due to the TREP-mediated effect on salivary gland entry and the slightly diminished capacity of *tlp*(-) sporozoites to migrate in the skin [40–42].

## Discussion

The availability of a positive–negative selection system has allowed the sequential deletion of genes in *P. berghei,* as transgenic parasite lines can be selected for parasites that lost the resistance cassette. In theory, this approach allows for an unlimited number of sequential genetic modifications. To test this capacity, three members of a family of adhesins that are expressed on *Plasmodium* sporozoites were targeted to create double and triple knockout lines, evaluating potential synergistic, additive, or antagonistic roles of these proteins. The analysis of the generated double and triple deletion parasites showed that the defect in adhesion and gliding of the sporozoites largely mimicked the defect of the dominant gene, e.g., a parasite lacking *trap* showed the same defect in terms of gliding and salivary gland invasion whether just *trap* was deleted or whether in addition *trep* or *tlp* or both were deleted. The only observed difference was that parasite lines lacking both *trap* and *trep* had a smaller percentage of waving sporozoites compared to lines in which only one of those genes was absent (Fig. 3A). It is not clear what physiological role waving plays in sporozoite biology in vivo, but in vitro it indicates the capacity of the sporozoite to attach to a surface. Hence, this observation suggests that TREP and TRAP both play a role in forming single adhesions sites and complements previous work implicating TREP (S6) in the cohesive strength of adhesion sites at the front of the parasite and TRAP at the rear end [43]. It is possible that TREP is secreted at the apex and allows sporozoites to form adhesions at the front-end enabling waving, which is followed by a second adhesion at the rear [43, 59]. Consequently, a lack of TREP weakening this first adhesion could lower the efficiency of the formation of a secondary adhesion site, a prerequisite of gliding. The observation that TREP is only important for salivary gland entry suggests that TREP is important for sporozoites to attach to salivary glands. TRAP could help in generating or stabilizing these adhesion patches, while mainly functioning in motility.

Only the mutant lacking both *trep* and *tlp* could enter into salivary glands in sufficient numbers to allow further experiments. The efficiency of *trep(-)/tlp(-)* sporozoites to invade salivary glands was comparable to *trep(-)* parasites. The reduction of infectivity of *trep(-)/tlp(-)* sporozoites transmitted by mosquito bite is likely a result of the decreased numbers in salivary glands as for *trep(-)* parasites [60]. In addition there might be an effect of slower migration in the skin, but this would need further experiments using sensitive in vitro assays such as our recently established 3D gel-based assay [27] or in vivo imaging, which would require fluorescent parasite lines and much time. Both experiments are nearly impossible to achieve in parasite lines with strongly reduced salivary gland loads. Intriguingly, *trep(-)/tlp(-)* salivary gland sporozoites showed a similar infectivity of mice as *tlp(-)* sporozoites when injected intravenously. This further suggests that a reduced number of transmitted sporozoites to the skin are the key reason for reduced by bite infection.

Taken together, no evidence was obtained of a synergistic or antagonistic effect of the investigated proteins. TRAP-family proteins appear to work independently of each other to enable sporozoite infection of salivary glands (TRAP, TREP) and efficient transmission to mice (TRAP, TLP). Interestingly, however, the observation that sporozoites lacking all three TRAP-family adhesins could still undergo some active motion, i.e., waving and patch gliding, suggests that additional proteins are important in sporozoite adhesion and the transition from adhesion to motility. It is important to note that sporozoites move in a stick–slip fashion that necessitates a continuous cycle of adhesion and deadhesion at distinct parts of the parasite [32]. Many other proteins are implicated in sporozoite gliding and among these are some that are likely to confer adhesive capacity to the sporozoite such as the complex PCRMP proteins [61], the circumsporozoite protein CSP and TRP1 [3]. These might contribute to modulating motility by influencing the adhesion–deadhesion cycle important for stick–slip locomotion. None of them were targeted here as deletion of *trp1* inhibits sporozoite egress from oocysts [3] and deletion of *csp* inhibits sporozoite formation [62]. Furthermore, the MAEBL and AMA1 proteins, involved in sporozoite invasion of the salivary gland and liver, respectively [63–65], could play a role, as well as some recently identified rhoptry proteins that are involved in gliding motility [66, 67].

Lastly, there are also other proteins with no transmembrane domain that are linked to adhesion and gliding motility, such as LIMP and CelTOS [68, 69]. While nothing is known about their mechanistic function, some link to intracellular proteins could be expected and they might link to all or selected members of the TRAP family.

## Conclusions

In conclusion, it was shown that the three TRAP-family members TRAP, TREP and TLP expressed in *Plasmodium* sporozoites can be deleted from the *P. berghei* genome with no effect on sporozoite formation and egress from oocysts. The observed phenotypes suggest that the three adhesins act independently from each other and that other proteins are also involved in sporozoite adhesion and motility. TRAP and TREP might be important for adhesion formation allowing sporozoite waving.

## Supplementary Information

**Additional file 1: Figure S1.** Generation of *tlp(-)/trap(-)* parasites via double homologous recombination. The cartoons **(A, B)** show the cloning strategy and primers used for genotyping with amplicon sizes of the resulting transgenic line indicated. Primer sequences are listed in table S2. The plasmid contains mCherry and the resistance marker yFCU for negative selection. **(C)** Resulting agarose gel picture after genotyping with the expected amplicon sizes given below. **(D)** Summary of primers and amplicon sizes for the genotyping PCR.

**Additional file 2: Figure S2.** Generation of *tlp(-)/trep(-)* parasites via double homologous recombination. The cartoon **(A, B)** shows the cloning strategy and primers used for genotyping with amplicon sizes of the resulting transgenic line indicated. Primer sequences are listed in table S2. The plasmid contains mCherry and the resistance marker yFCU for negative selection. **(C)** Resulting Agarose gel picture after genotyping with the expected amplicon sizes given below. **(D)** Summary of primers and amplicon sizes for the genotyping PCR.

**Additional file 3: Figure S3.** Generation of *trap(-)/trep(-)* parasites via double homologous recombination. The cartoon **(A, B)** shows the cloning strategy and primers used for genotyping with amplicon sizes of the resulting transgenic line indicated. Primer sequences are listed in table S2. Plasmid contains mCherry and the resistance marker yFCU for negative selection. **(C)** Resulting agarose gel picture after genotyping with the expected amplicon sizes given below. **(D)** Summary of primers and amplicon sizes for the genotyping PCR.

**Additional file 4: Figure S4.** Generation of *tlp(-)/trep(-)/trap(-)* parasites via double homologous recombination. The cartoon **(A, B, C)** shows the cloning strategy and primers used for genotyping with amplicon sizes of the resulting transgenic line indicated. Primer sequences are listed in table S2. The plasmid contains mCherry and the resistance marker yFCU for negative selection. **(D)** Resulting agarose gel picture after genotyping with the expected amplicon sizes given below. **(E)** Summary of primers and amplicon sizes for the genotyping PCR.

**Additional file 5: Table S1.** Primer list

## Data Availability

All generated data are displayed within the manuscript and generated parasite lines are available from the corresponding author on request.

## Abbreviations

CelTOS: Cell-traversal protein for ookinetes and sporozoites
CSP: Circumsporozoite protein
PCRMP: Plasmodium cysteine repeat modular proteins
CTRP: Circumsporozoite- and TRAP-related protein
S6: Sporozoite-specific protein 6
TLP: TRAP-like protein
TRAP: Thrombospondin related anonymous protein
TREP: TRAP-related protein
TRP1: Thrombospondin related protein 1
UOS3: Upregulated in oocyst sporozoites protein 3
