# Supplementary figures and images for: Limited Plasmodium sporozoite gliding motility in the absence of TRAP family adhesins
Source: Malar J. 2021 Oct 30;20:430. doi: 10.1186/s12936-021-03960-3 (PMC8557484; doi:10.1186/s12936-021-03960-3)

# Figure S1

## Generation of TLP(-)/TRAP(-) parasites

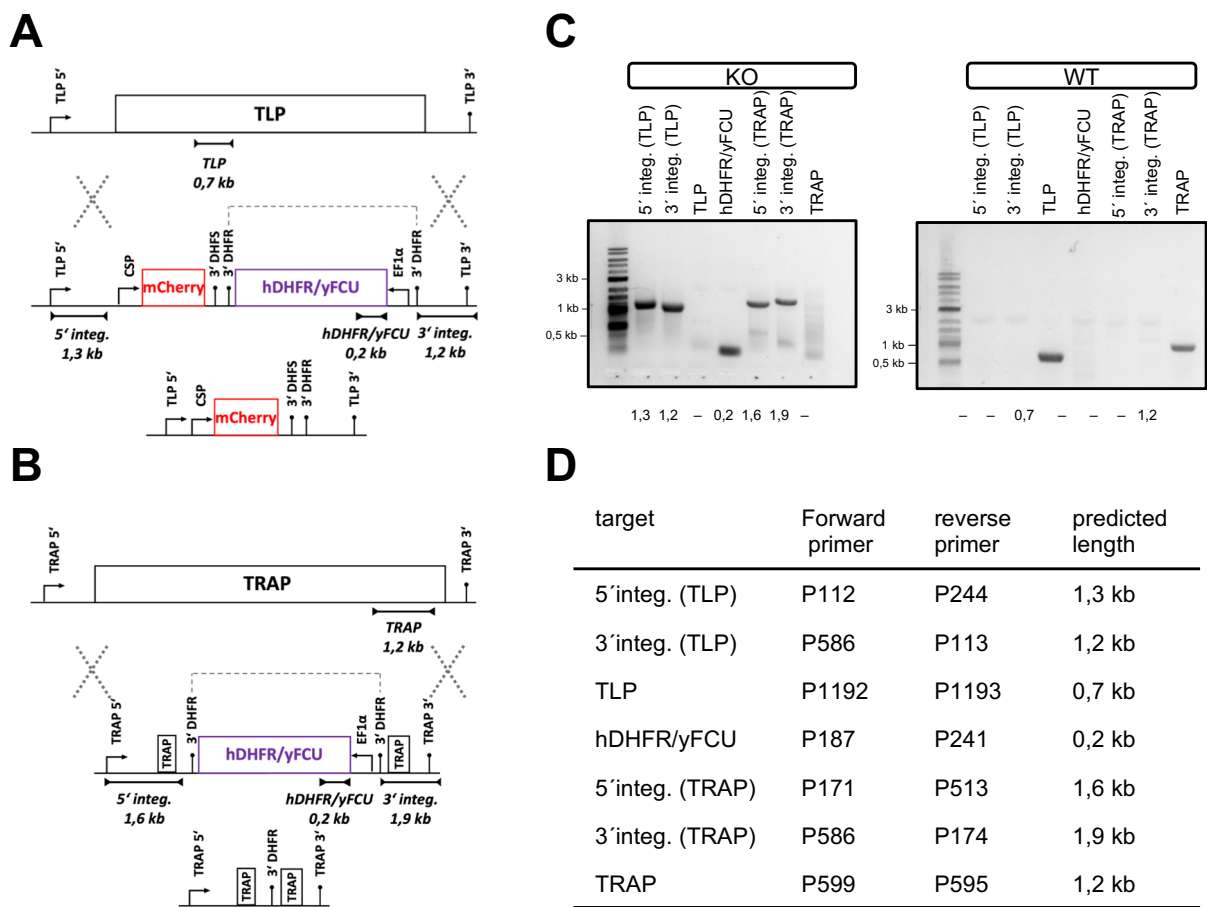

Supplement: Supplementary file 1 — Additional file 1: Figure S1. Generation of tlp(-)/trap(-) parasites via double homologous recombination. The cartoons (A, B) show the cloning strategy and primers used for genotyping with amplicon sizes of the resulting transgenic line indicated. Primer sequences are listed in table S2. The plasmid contains mCherry and the resistance marker yFCU for negative selection. (C) Resulting agarose gel picture after genotyping with the expected amplicon sizes given below. (D) Summary of primers and amplicon sizes for the genotyping PCR. [file 12936_2021_3960_MOESM1_ESM.pdf]

**Figure S2**  
**Generation of TLP(-)/TREP(-) parasites**

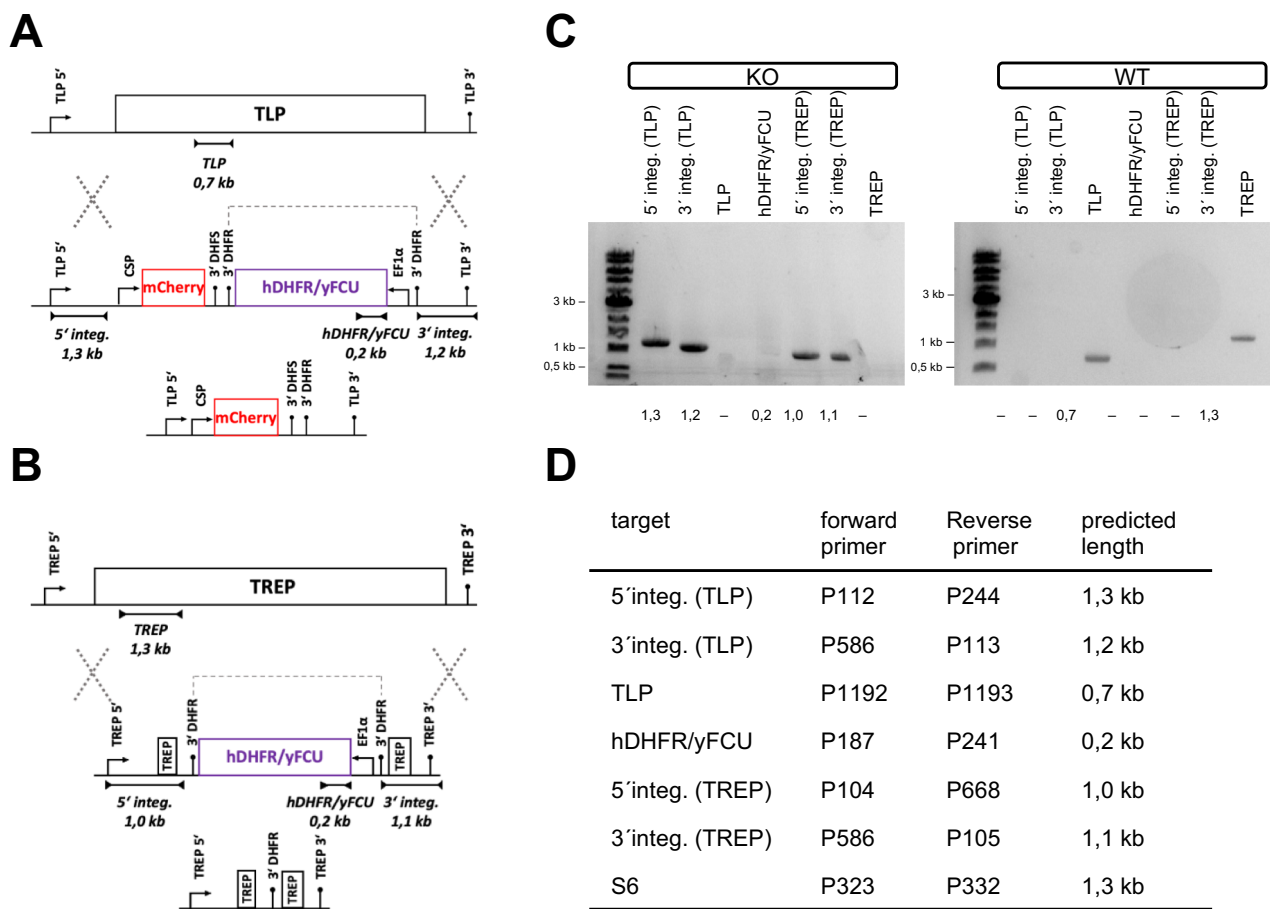

Supplement: Supplementary file 2 — Additional file 2: Figure S2. Generation of tlp(-)/trep(-) parasites via double homologous recombination. The cartoon (A, B) shows the cloning strategy and primers used for genotyping with amplicon sizes of the resulting transgenic line indicated. Primer sequences are listed in table S2. The plasmid contains mCherry and the resistance marker yFCU for negative selection. (C) Resulting Agarose gel picture after genotyping with the expected amplicon sizes given below. (D) Summary of primers and amplicon sizes for the genotyping PCR. [file 12936_2021_3960_MOESM2_ESM.pdf]

# Figure S3

## Generation of TREP(-)/TRAP(-) parasites

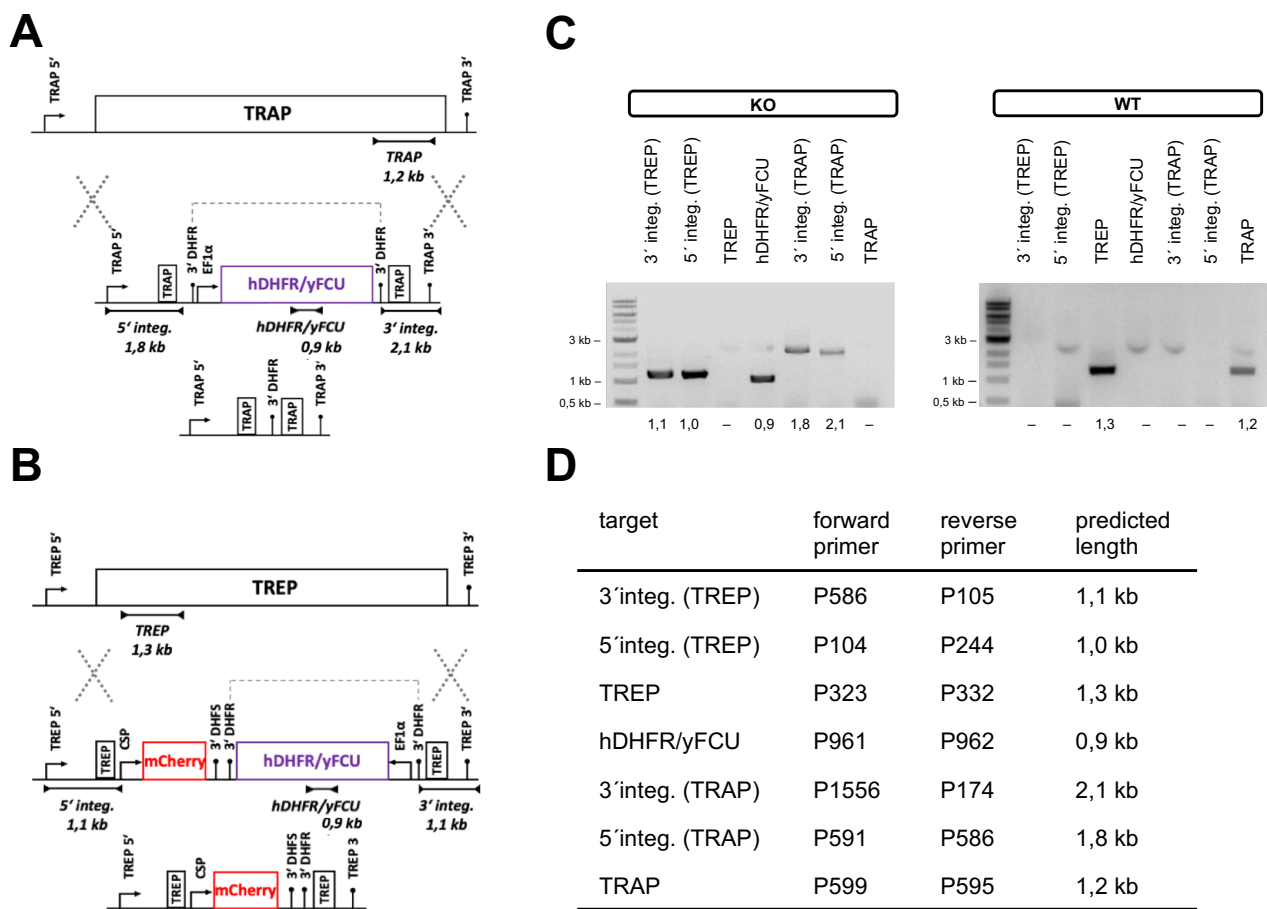

Supplement: Supplementary file 3 — Additional file 3: Figure S3. Generation of trap(-)/trep(-) parasites via double homologous recombination. The cartoon (A, B) shows the cloning strategy and primers used for genotyping with amplicon sizes of the resulting transgenic line indicated. Primer sequences are listed in table S2. Plasmid contains mCherry and the resistance marker yFCU for negative selection. (C) Resulting agarose gel picture after genotyping with the expected amplicon sizes given below. (D) Summary of primers and amplicon sizes for the genotyping PCR. [file 12936_2021_3960_MOESM3_ESM.pdf]

# Figure S4

## Generation of TLP(-)/TREP(-)/TRAP(-) parasites

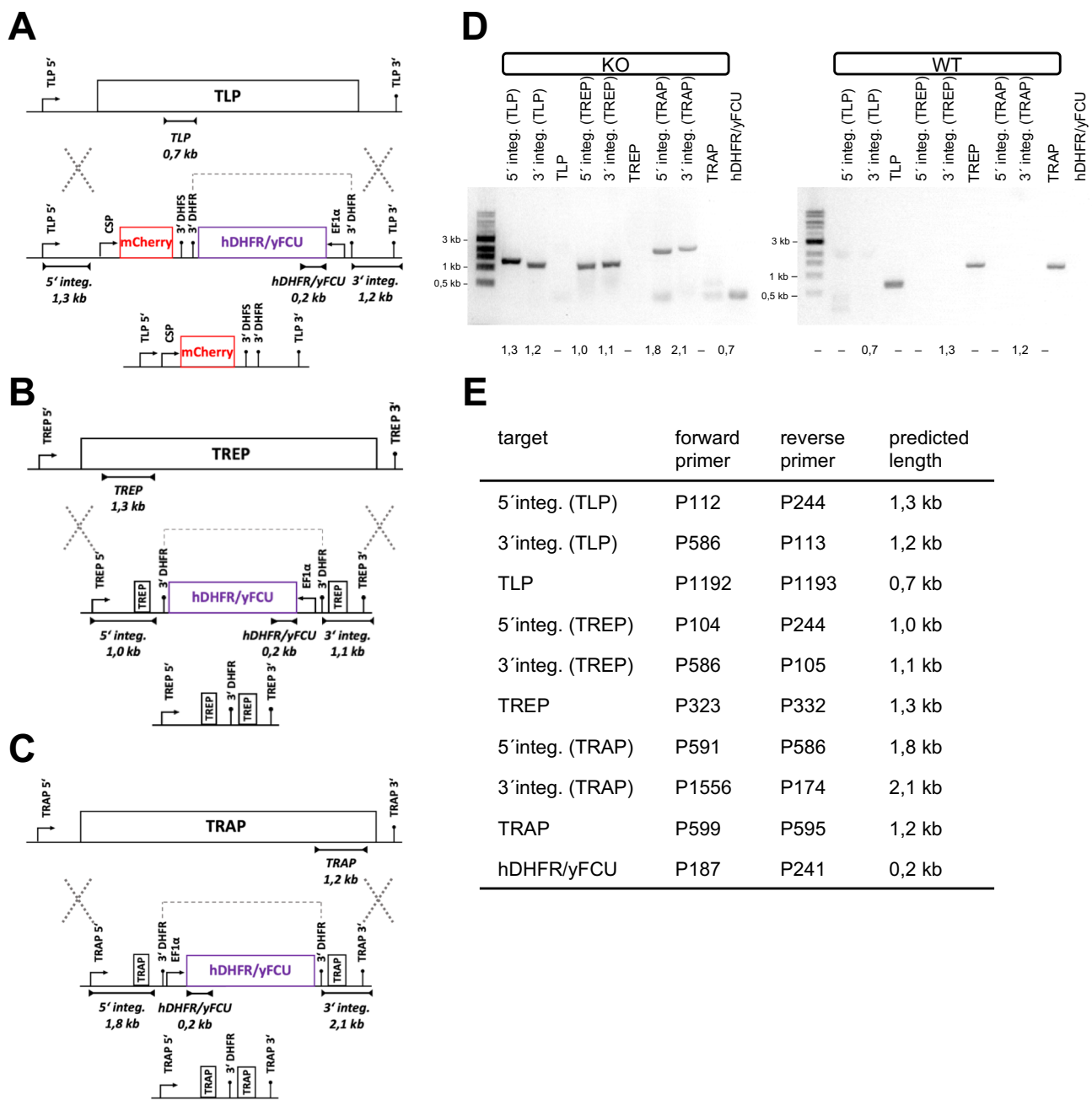

Supplement: Supplementary file 4 — Additional file 4: Figure S4. Generation of tlp(-)/trep(-)/trap(-) parasites via double homologous recombination. The cartoon (A, B, C) shows the cloning strategy and primers used for genotyping with amplicon sizes of the resulting transgenic line indicated. Primer sequences are listed in table S2. The plasmid contains mCherry and the resistance marker yFCU for negative selection. (D) Resulting agarose gel picture after genotyping with the expected amplicon sizes given below. (E) Summary of primers and amplicon sizes for the genotyping PCR. [file 12936_2021_3960_MOESM4_ESM.pdf]
