# Supplementary material for: Limited Plasmodium sporozoite gliding motility in the absence of TRAP family adhesins
Source: Malar J. 2021 Oct 30;20:430. doi: 10.1186/s12936-021-03960-3 (PMC8557484; doi:10.1186/s12936-021-03960-3)
Supplement: Supplementary file 5 — Additional file 5: Table S1. Primer list [file 12936_2021_3960_MOESM5_ESM.docx]

**Table S1**

**Primer list**

P104: GGCTTTTAGCATTTTATTAACAATCG

P105: GGATAATCATTTTTTTCACCTGAAGC

P112: TTTTGAGAAGGTATAACCCATATTCC

P113: TCCCCGCGGAACATCCATATTAAATAACATCG

P171: GAATACATGTAAAAAAGAGAAATTCCTTCG

P174: GTAAAATAAGCGATATAGAAGGGAGC

P187: TTCTACTGAAGAGGTTGTGGTC

P241: CTTGCACCGGTATGGTTGGTTCGCTAAAC

P244: ACGCAACCTTATATTCCTCAATTAC

P323: TAAAGCACTTAATATATGCGATTATGGG

P332: CCGGATATCCCATCTTTATGGGCTTCGC

P513: GAGTTTAAACCTGTAAAAATGTGTATGTTGTGTGC

P586: CATACTAGCCATTTTATGTG

P591: TCGCTTTAACGGTTGATGCA

P595: GCCATCAGGGGGTGCAGAACC

P599: TCGCTGCATTAATGATTTGCCT

P600: CCCAAGCTTCAAAAAAGCAGGCTTGCCGC

P601: GCCGATATCCAAGAAAGCTGGGTGGTACCC

P668: TGATTAGCATAGTTAAATAAAAAAAGTTG

P1192: ACGCGTCGACGATAATATCGATACAGACCCTTATTGG

P1193: GATATCATTATTATTATTATGATCTATATATGATTCAATACCTTTAT

TATTTC

P1556: CCCACCGGTTACCAATTGATTGTATTTATAACTGTAAAAATGTG
